# A Single-Tube Two-Step MIRA-CRISPR/Cas12b Assay for the Rapid Detection of Mpox Virus

**DOI:** 10.3390/v17060841

**Published:** 2025-06-12

**Authors:** Ge Hu, Zhijie Wei, Jinlei Guo, Kangchen Zhao, Qiao Qiao, Xiaojuan Zhu, Tao Wu, Heng Rong, Shuo Ning, Ziyang Hao, Ying Chi, Lunbiao Cui, Yiyue Ge

**Affiliations:** 1 School of Public Health, Nanjing Medical University, Nanjing 211166, China; huge20232023@163.com (G.H.); 19159287191@163.com (Z.W.); haoziy2499@163.com (Z.H.); 2 NHC Key Laboratory of Enteric Pathogenic Microbiology, Jiangsu Provincial Medical Key Laboratory of Pathogenic Microbiology in Emerging Major Infectious Diseases, Jiangsu Provincial Center for Disease Control and Prevention, Nanjing 210009, China; jinleiguo0708@163.com (J.G.); zhaokangchen@jscdc.cn (K.Z.); qiaoqiao@jscdc.cn (Q.Q.); zhuxiaojuan@jscdc.cn (X.Z.); wutao@jscdc.cn (T.W.); rh@jscdc.cn (H.R.); ningshuo0620@163.com (S.N.); chiying@jscdc.cn (Y.C.)

**Keywords:** Mpox, MIRA, CRISPR/Cas12b, tricosane

## Abstract

Mpox is a zoonotic disease caused by the Mpox virus (MPXV). The rapid and accurate diagnosis of MPXV is essential for the timely and effective prevention, control, and treatment of the disease. In this study, we combined Multienzyme Isothermal Rapid Amplification (MIRA) (at 42 °C) and Clustered Regularly Interspaced Short Palindromic Repeats/CRISPR-associated protein 12b(CRISPR/Cas12b) (at 60 °C) to develop a single-tube two-step assay for rapid MPXV detection, leveraging the distinct physical states of tricosane at these temperatures. MIRA amplification primers and CRISPR/cas12b SgRNA were designed based on the MPXV F3L gene. After screening the primers and sgRNAs, the reaction conditions were optimized, and the performances of the assay were evaluated. The detection limit (LOD) of this single-tube two-step MIRA-CRISPR/Cas12b assay for MPXV is four copies of DNA molecules. No cross-reactivity with other pathogens (herpes simplex virus (HSV), Epstein–Barr virus (EBV), Coxsackievirus A16 (CVA16), Enterovirus A71 (EV-A71), and measles virus (MeV)) was found. The assay also showed good consistency with quantitative real-time PCR (qPCR) (Kappa = 0.9547, *p* < 0.05, *n* = 100) in the detection of clinical samples, with a sensitivity of 98.5% and a specificity of 97.0%. The single-tube two-step MIRA-CRISPR/Cas12b assay permits the rapid (within 45 min), sensitive, and specific detection of MPXV. The lack of need for opening the reaction tube eliminates the risk of product contamination.

## 1. Introduction

Mpox virus (MPXV) is classified into two clades: Central African (clade I) and West African (clade II) [1,2]. Clade I is more virulent, with a case fatality rate as high as 10% in the absence of smallpox vaccination [3]. Since the United Kingdom reported cases of human MPXV infection (with the predominant strain being type IIb) in 2022, the Mpox outbreak has rapidly spread to regions in Europe, the Americas, and Asia. In September 2023, a variant of MPXV clade I, designated as type Ib, emerged in the Democratic Republic of the Congo and has since gradually spread to neighboring countries [4]. As of now, there have been approximately over 120,000 confirmed cases of Mpox infection globally, with 676 reported deaths [5]. The Mpox outbreak poses a significant threat to human health and socio-economic development.

The rapid and accurate diagnosis of the MPXV is crucial for the timely implementation of effective containment measures and the early selection of appropriate therapeutic agents. Current laboratory diagnostic methods for MPXV include immunological assay [6] and nucleic acid testing (NAT) [7,8]. Compared with NATs, immunological assays exhibit lower sensitivity and specificity [9]. Among NATs, quantitative real-time PCR (qPCR) is currently the primary laboratory diagnostic method for MPXV due to its high sensitivity and specificity [10]. However, nucleic acid isothermal amplification (NAIA) technology has been gradually and widely adopted because of its advantages, such as high amplification efficiency, fast speed, and low cost.

The Clustered Regularly Interspaced Short Palindromic Repeats/CRISPR-associated protein (CRISPR/Cas) system is an adaptive immune system originating from bacteria and archaea. In recent years, researchers have harnessed the trans-cleavage properties of Class II Cas proteins to integrate the CRISPR/Cas system with NAIA for applications in nucleic acid detection [11]. Currently, the representative CRISPR technologies for pathogen detection are the Specific High-Sensitivity Enzymatic Reporter Unlocking (SHERLOCK), based on Cas13a [11], and the DNA Endonuclease-Targeted CRISPR Trans Reporter (DETECTR), based on Cas12a [12]. The target nucleic acids are first amplified using NAIA techniques, followed by detection via the trans-cleavage activity of Cas proteins. However, as the enrichment and detection of the target are conducted in separate steps, multiple tube openings during the experimental process posed a risk of aerosol contamination [13]. The one-step method, such as the SHERLOCK Testing in One Pot (STOP) [14] technique, combines all reaction components into a single mixture and employs simultaneous amplification and detection. This approach mitigates the aerosol contamination risk associated with tube opening in the traditional two-step SHERLOCK method. However, the CRISPR/Cas cleavage reaction affects the accumulation of NAIA products, and the two reaction systems are not fully compatible, resulting in suboptimal detection sensitivity of the one-step NAIA-CRISPR/Cas detection system [15]. Therefore, it is necessary to develop a technology that can achieve high-sensitivity two-step detection without the need to open the reaction tube.

Multienzyme Isothermal Rapid Amplification (MIRA) is an isothermal amplification technique similar to RPA. It relies on the coordinated action of multiple functional proteins (such as helicase, recombinase, single-strand binding protein, DNA polymerase, etc.) to achieve rapid nucleic acid amplification at room temperature. By integrating MIRA technology, which operates at temperatures ranging from 37 to 42 °C, with the AapCas12b nuclease (C2c1) that functions optimally between 58 and 65 °C, we have developed a novel single-tube two-step assay for MPXV. To achieve a single-tube two-step assay without the need for tube opening, by leveraging the distinct physical states of tricosane at different temperatures, this study employs it as a physical barrier between separate reaction systems. Tricosane is an insoluble solid at room temperature, and it melts to form a liquid when the temperature exceeds 46 °C; therefore, it remains in a solid state during the MIRA reaction to physically separate the reaction systems while transitioning to a liquid phase during the Cas12b reaction to allow for the complete mixing of the reaction components. This approach not only prevents aerosol contamination but also maintains the sensitivity of the assay. Compared with qPCR, it achieves faster detection, with the entire process completed within 45 min. The experimental results can be read using an isothermal fluorescence detector or by the naked eye, without the need for complex equipment. Moreover, this method allows for the simultaneous detection of clade II and clade I viruses, offering a novel, rapid, and sensitive approach for the detection of MPXV.

## 2. Materials and Methods

### 2.1. Virus Strains and Clinical Samples

The MPXV strains were isolated in the Biosafety Level-3 (BSL-3) laboratory of the Jiangsu Provincial Center for Disease Control and Prevention. A total of 100 clinical samples (including serum, throat swabs, and vesicle fluid) were obtained from individuals suspected to be infected with MPXV in Jiangsu Province.

### 2.2. Ethical Statement

This study was approved by the ethical Review Committee of the Jiangsu Provincial Center for Disease Control and Prevention (JSJK2025-B005-01). All the experiments were performed in accordance with the Declaration of Helsinki.

### 2.3. Primer, Probe, and Single-Guide RNA (sgRNA) Design

A total of 56 representative full-length MPXV genome sequences were downloaded from the NCBI GenBank database. These sequences were aligned, and their highly conserved regions were analyzed. The conserved region of the F3L segment was selected for the design of the forward and reverse amplification primers for MIRA. The primers were evaluated using Oligo 7 software. The sgRNA was designed based on the F3L region of MPXV. The sequence of the fluorescent probe used for Cas12b detection was referred to from reference [16]. All primers, probes, and sgRNA in vitro transcription templates were synthesized by Sangon Biotech (Shanghai, China), and the specific sequences are shown in Table 1.

### 2.4. sgRNA Preparation

The lyophilized sgRNA in vitro transcription template was reconstituted with water to a concentration of 500 ng/µL to prepare the pre-transcription template. A reaction mixture was assembled by combining 1 µL of the 500 ng/µL pre-transcription template, 5 µL of 10× T7 Buffer (Takara Bio, Kusatsu, Japan), 1 µL of T7 primer(5′-TAATACGACTCACTATAGGG-3′), and 30 µL of nuclease-free water. This mixture was subjected to a two-step reaction: First, incubation at 95 °C for 30 s, followed by incubation at 25 °C for 2 min, was performed. Subsequently, 5 µL of 50 mM DTT (Takara Bio, Kusatsu, Japan), 4 µL of 25 mM NTPs mix (Takara Bio, Kusatsu, Japan), 1.25 µL of Recombinant RNase inhibitor (Takara Bio, Kusatsu, Japan), and 2.5 µL of T7 RNA polymerase (Takara Bio, Kusatsu, Japan) were added to the mixture, and the reaction was carried out at 42 °C for 2 h. Finally, 5.8 µL of 10× DNase I Buffer (Takara Bio, Kusatsu, Japan) and 2 µL of Recombinant DNase I (Takara Bio, Kusatsu, Japan) were added to the reaction mixture, which was then incubated at 37 °C for 1 h and subsequently incubated at 80 °C for 2 min. The in vitro transcribed sgRNA was purified following the protocol provided in the manual for the Spin Column RNA Rapid Concentration and Purification Kit (Sangon Biotech, Shanghai, China). After purification, the concentration of the sgRNA was measured using the Qubit™ RNA BR Assay Kit (Thermo Fisher, Waltham, MA, USA) and a Qubit FLEX fluorometer (Thermo Fisher, Waltham, MA, USA). The prepared sgRNA was stored at −80 °C.

### 2.5. Tricosane Microsphere Preparation

Solid tricosane was melted into a liquid by heating at 60 °C for 30 s. Subsequently, the liquid was drawn up using a pipette and dropped onto a glass slide (15 µL per drop). The drops were then cooled and solidified into spherical shapes at room temperature. All preformed microspheres were stored at room temperature.

### 2.6. Establishment of the Single-Tube Two-Step MIRA-CRISPR/Cas12b Detection Procedure

**Figure 1:**
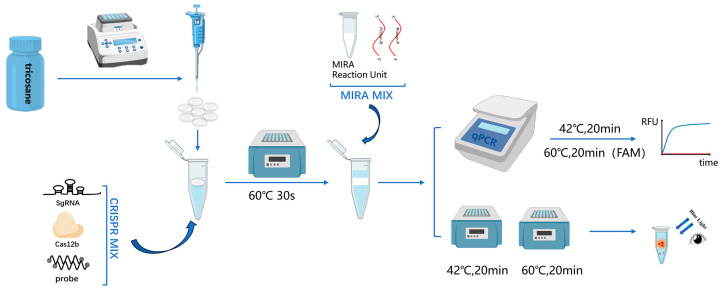
Schematic diagram of the single-tube two-step MIRA-CRISPR/Cas12b detection procedure.

The single-tube two-step MIRA-CRISPR/Cas12b detection procedure consists of two steps. In the first step, a 10 µL MIRA amplification reaction system (Cat#: WLB8201KIT, Amp-Future Biotech Co., Ltd., Changzhou, China) is prepared, which includes the MIRA basic reaction unit, 6 µL of Buffer A, 0.8 µL of forward primer, 0.8 µL of reverse primer, 1 µL of enzyme-free water, 0.4 µL of Buffer B, and 1 µL of template. The second step is the CRISPR/Cas12b detection reaction, which is configured according to the instructions provided with the AapCas12b (C2c1) Nuclease kit (Cat#: 32118, Shanghai Tolo Biotech Co. Ltd., China). The CRISPR/Cas12b detection system comprises 4 µL of 10× HOLMES Buffer, 1.0 µL of 10 µM AapCas12b Nuclease, 1 µL of 10 µM sgRNA, 1 µL of 10 µM ssDNA Reporter, 23 µL of nuclease-free water, and 10 µL of MIRA amplification product. Initially, the Cas12b detection system was added to the bottom of the reaction tube. Subsequently, preformed tricosane microspheres were added. The tube was heated to 60 °C to melt the tricosane, which solidified to form a barrier above the Cas12b system when it was cooled to room temperature. Then, the pre-assembled MIRA system was added to the barrier. For fluorescence detection, the reaction was performed on the qPCR instrument with the following program: 20 min at 42 °C, followed by 20 min at 60 °C (collection of FAM fluorescence at this step, with data acquisition every 30 s). For visual detection, the reaction was carried out in a constant-temperature metal bath under the same conditions of 20 min at 42 °C and 20 min at 60 °C. After the reaction, the results were visually interpreted using irradiation with 485 nm blue light. The whole process is shown in Figure 1.

### 2.7. Nucleic Acid Extraction

RNA was extracted from 200 μL of each sample or virus strains (inactivated in the BSL-3 laboratory) on an automatic nucleic acid extraction instrument GeneRotex 96 (Tianlong, Xi’an, China) with a Virus DNA/RNA Extraction Kit (Tianlong, Xi’an, China) according to the manufacturer’s instructions. The extracted nucleic acids were kept at −80 °C.

### 2.8. Sensitivity

The nucleic acid of MPXV strains (hMpxV/China/Jiangsu/23001 (type IIb C.1)) was quantitatively analyzed using the digital PCR method by using QIAcuity One (QIAGEN, Hilden, Germany) and a QIAcuity Probe PCR Kit (Cat#: 250101, QIAGEN, Hilden, Germany). The MPXV nucleic acid was serially diluted (10^4^, 10^3^, 10^2^, 10^1^, and 10^0^ copies/µL) and used as templates to evaluate the sensitivity of the single-tube two-step MIRA-CRISPR/Cas12b assay. Eight parallel tests were performed on each dilution to determine the limit of detection (LOD) of the assay. Meanwhile, the conventional two-step method (without the usage of tricosane) was also carried out on each dilution to compare the detection performance between the single-tube two-step MIRA-CRISPR/Cas12b assay and the traditional two-step assay.

### 2.9. Specificity

Nucleic acids from viruses that exhibit symptoms similar to MPXV infection were selected as templates to evaluate the specificity of the single-tube two-step MIRA-CRISPR/Cas12b detection system. These included herpes simplex virus (HSV), Epstein–Barr virus (EBV), Coxsackievirus A16 (CVA16), Enterovirus A71 (EV-A71), and measles virus (MeV). All these viruses were obtained from the routine infectious disease surveillance program of the Jiangsu Provincial Center for Disease Control and Prevention, with each virus confirmed by qPCR.

### 2.10. Validation with Clinical Samples

Nucleic acids were extracted from 100 clinical samples. Both qPCR and the single-tube two-step MIRA-CRISPR/Cas12b assay were employed for parallel testing. The results from qPCR were used as a reference to evaluate the accuracy of the single-tube two-step MIRA-CRISPR/Cas12b assay in testing clinical samples.

### 2.11. Statistical Analysis

Statistical analyses were performed using IBM SPSS Statistics 26 software. The LOD of the assay was determined by probit regression analysis. The level of agreement between the results obtained by the single-tube two-step MIRA-CRISPR/Cas12b assay and qPCR was assessed using the Kappa statistic.

## 3. Results

### 3.1. Optimization of Tricosane Volume in the Single-Tube Two-Step MIRA-CRISPR/Cas12b Assay

**Figure 2:**
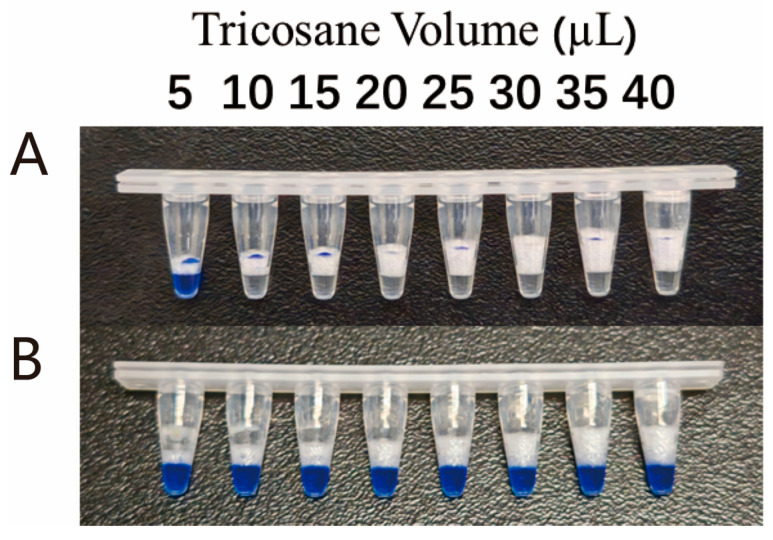
Optimization of tricosane volume. (**A**) Blocking effect of different volumes of tricosane at 42 °C; (**B**) Blocking effect of different volumes of tricosane at 60 °C.

Initially, the blocking effect of tricosane at 42 °C was evaluated. A total of 10 µL blue Gold View solution was used as a substitute for the 10 µL amplification product to simulate the blocking effect of different volumes of tricosane at 42 °C. As shown in Figure 2A, when the volume of tricosane was ≥15 µL, it completely prevented the diffusion of the upper layer into the lower layer. Subsequently, when the reaction tube was heated to 60 °C, which is required for the CRISPR/Cas12b reaction, as shown in Figure 2B, the upper layer of liquid completely entered the lower part of the reaction tube and mixed with the CRISPR/Cas12b detection system. Therefore, a volume of 15 µL was selected as the optimal volume for the addition of tricosane.

### 3.2. Optimization of the Detection System and Reaction Conditions for the Single-Tube Two-Step MIRA-CRISPR/Cas12b Assay

**Figure 3:**
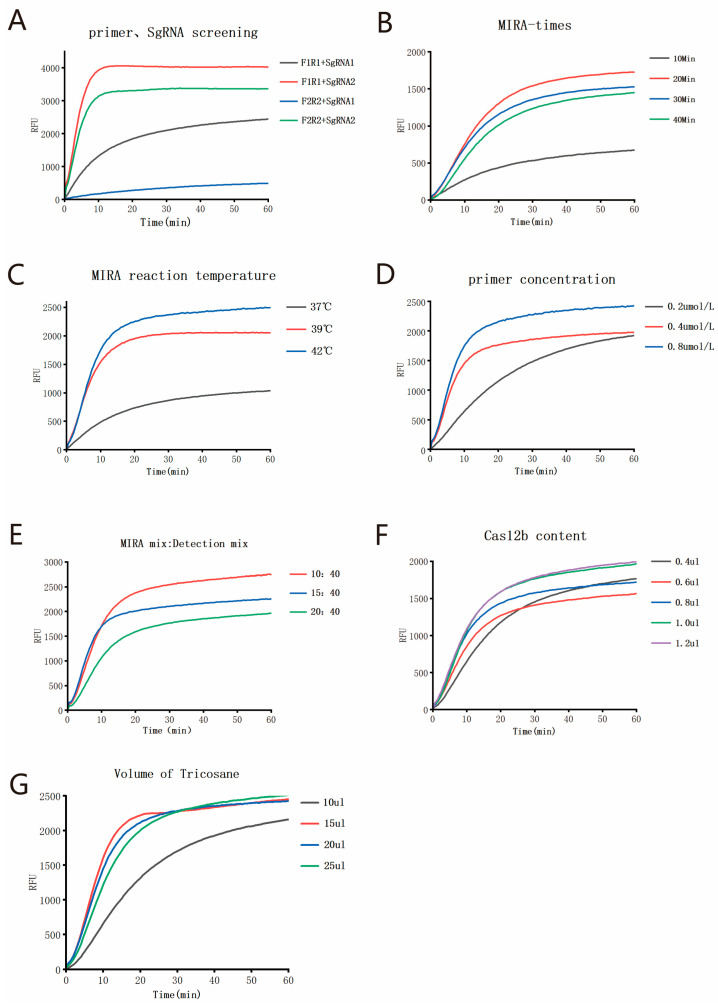
Optimization of the detection system and reaction conditions for the single-tube two-step MIRA-CRISPR/Cas12b assay: (**A**) Screening of MPXV primers and sgRNA; (**B**) Optimization of MIRA reaction time; (**C**) Optimization of MIRA reaction temperature; (**D**) Optimization of final primer concentration in MIRA reaction; (**E**) Optimization of the volume ratio of MIRA amplification reaction to CRISPR/Cas detection reaction; (**F**) Optimization of Cas12b concentration.; (**G**) Optimization of tricosane volume.

Initially, the nucleic acid of the MPXV strain was used as the template for MIRA amplification. Different combinations of MIRA amplification primers and sgRNAs were screened. The results of the MIRA-CRISPR/Cas12b detection are shown in Figure 3A. Among these primer pairs and sgRNA combinations, the combination of F1R1 and sgRNA2 exhibited the earliest amplification peak and the highest amplification efficiency. Therefore, this combination was selected as the optimal primer pair and sgRNA for subsequent experiments. Subsequently, using 100 copies/µL MPXV nucleic acid as templates, the MIRA reaction time, reaction temperature, and final primer concentration were optimized. Initially, following the instructions provided with the MIRA nucleic acid amplification kit and the TOLOBIO AapCas12b Nuclease kit, the final concentration of the MIRA amplification primers was fixed at 0.4 µmol/L, the reaction temperature was fixed at 42 °C, the volume of 10 µM AapCas12b Nuclease was fixed at 1 µL, and the volume ratio of the MIRA amplification reaction to the CRISPR/Cas12b detection reaction was fixed at 10:30 to optimize the optimal reaction time of MIRA. As shown in Figure 3B, except for the relatively low signal when the reaction time was set at 10 min, high fluorescence signals were obtained at 20 min, 30 min, and 40 min. Therefore, 20 min was determined to be the optimal MIRA reaction time. Then, with the MIRA reaction time fixed at 20 min, the MIRA reaction temperature was optimized. As shown in Figure 3C, the strongest fluorescence appeared at 42 °C, which was thus selected as the optimal temperature for MIRA amplification. Subsequently, the final concentration of MIRA primers was optimized. The detection results are shown in Figure 3D. When the final concentration of primers was 0.8 µmol/L, the highest fluorescence signal appeared in the shortest time. Therefore, 0.8 µmol/L was ultimately chosen as the optimal final concentration for the MIRA amplification primers.

After that, the volume ratio of the MIRA amplification reaction to the CRISPR/Cas12b detection reaction was optimized. As shown in Figure 3E, the highest fluorescence signal was achieved when the volume ratio was 10:30. Finally, different amounts of Cas12b were added to the detection system. As shown in Figure 3F, as the amount of Cas12b increased, the fluorescence signal also increased. However, when the amount of Cas12b exceeded 1 µL, there was little change in the fluorescence signal in the shortest time, and the differences in detection were not statistically significant. Therefore, a final volume of 1 µL of Cas12b was selected as the optimal amount. According to the optimized reaction conditions, we used the actual amplification detection system to optimize the volume of tricosane used once again. The results showed that tricosane with a volume of 15 µL or more could also achieve the best fluorescence signal results, which was consistent with the results of the Gold View experiment (Figure 3G). Therefore, in subsequent studies, 15 µL of tricosane was used for the physical isolation of the reaction system.

### 3.3. Sensitivity Assay of the Single-Tube Two-Step MIRA-CRISPR/Cas12b Assay

**Figure 4:**
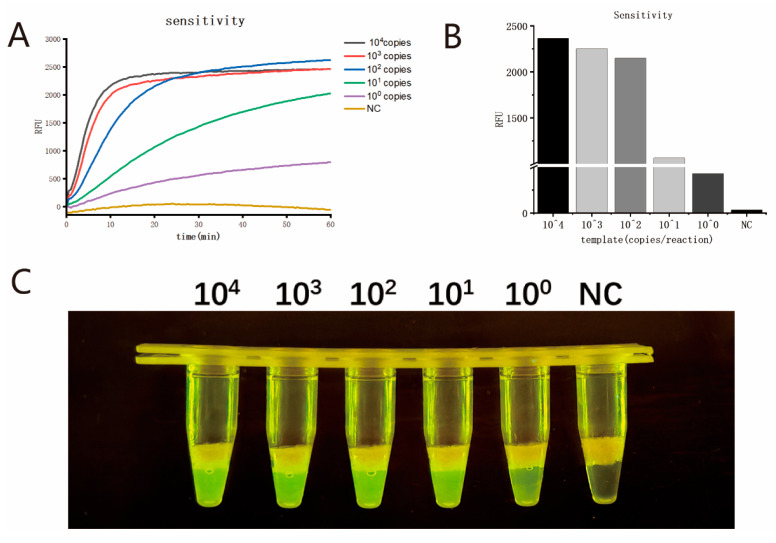
(**A**) Sensitivity assay of the single-tube two-step MIRA-CRISPR/Cas12b assay; (**B**) Fluorescence signal values in the sensitivity assay of the single-tube two-step MIRA-CRISPR/Cas12b assay after a 20 min reaction; (**C**) Visual detection sensitivity assay of the single-tube two-step MIRA-CRISPR/Cas12b assay after a 20 min reaction.

The digital PCR quantification results showed that the nucleic acid concentration of MPXV strains (hMpxV/China/Jiangsu/23001 (type IIb C.1)) was 1.4 × 10^4^ copies/μL. Then, the nucleic acid was serially diluted (10^4^, 10^3^, 10^2^, 10^1^, and 10^0^ copies/μL) and used as the template for the MIRA-CRISPR/Cas12b assay. The reactions were performed under optimized conditions. As shown in Figure 4A, after 20 min of the CRISPR/Cas12b reaction, a clear difference in fluorescence signal was observed. The fluorescence signal values after 20 min of the CRISPR/Cas12b detection reaction are shown in Figure 4B. The reaction tubes were then irradiated with blue light, and the detection results were visually observed, as shown in Figure 4C. The results were consistent with those obtained via fluorescence detection, with a detection sensitivity of one copy per reaction.

To further determine the LOD of this assay, eight replicate experiments were performed for each dilution series (10^4^, 10^3^, 10^2^, 10^1^, and 10^0^ copies) of MPXV template, with the CRISPR/Cas12b reaction time set at 20 min. The results are shown in Table 2. Probit regression analysis was applied to the data, yielding an LOD of four copies per reaction for this assay. The LOD of the traditional two-step MIRA-CRISPR/Cas12b assay is 3.4 copies per reaction.

### 3.4. Specificity of the Single-Tube Two-Step MIRA-CRISPR/Cas12b Assay

**Figure 5:**
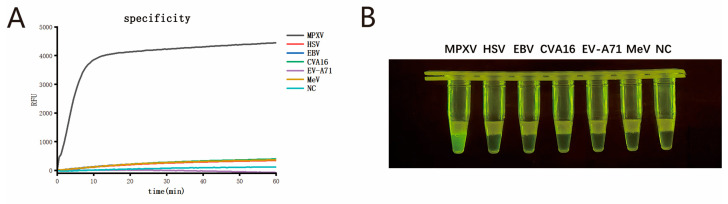
(**A**) Specificity assay of the single-tube two-step MIRA-CRISPR/Cas12b assay; (**B**) Visual detection specificity assay of the single-tube two-step MIRA-CRISPR/Cas12b assay.

To validate the specificity of the single-tube two-step MIRA-CRISPR/Cas12b assay for MPXV, we tested five other pathogens that present clinically similar to Mpox, including HSV, EBV, CVA16, EVA71, and MeV. The fluorescence detection results are shown in Figure 5A, and the visual detection results are shown in Figure 5B. No cross-reactivity was observed between MPXV and HSV, EBV, CVA16, EV-A71, or MeV, indicating that the established single-tube two-step MIRA-CRISPR/Cas12b assay exhibits good specificity.

### 3.5. Evaluation of the Detection Effectiveness in Clinical Samples

Nucleic acids were extracted from 100 clinical samples using an automated nucleic acid extractor. Both qPCR and the single-tube two-step MIRA-CRISPR/Cas12b method were employed for parallel testing. The results from qPCR were used as a reference to evaluate the accuracy of the single-tube two-step MIRA-CRISPR/Cas12b method in testing clinical samples. The detection results are shown in Table 3. The sensitivity and specificity of the single-tube two-step MIRA-CRISPR/Cas12b method were 98.5% (66/67) and 97.0% (32/33), respectively, with a positive predictive value of 98.5% (66/67) and a negative predictive value of 97.0% (32/33). The two detection methods exhibited good consistency (Kappa = 0.9547, *p* < 0.05, *n* = 100).

## 4. Discussion

Mpox is a zoonotic infectious disease caused by MPXV that is primarily transmitted through close contact, such as exposure to bodily fluids, broken skin, or contaminated bedding and clothing [2]. Infection with Mpox manifests as fever, headache, lymphadenopathy, and characteristic rashes. Additionally, Mpox infection may lead to a range of severe complications, including secondary bacterial infections of skin lesions and, in severe cases, bacterial sepsis, which can be fatal [17,18]. Since 2022, Mpox has been reported in multiple countries and regions globally, posing a serious threat to public health and safety. Therefore, the early and rapid detection of the Mpox virus is of great significance for the prevention and control of Mpox outbreaks. MIRA amplification technology, a rapid NAIA technique, can achieve exponential amplification of the target in 20 min at a constant temperature and has been widely used for pathogen detection [19]. In recent years, nucleic acid detection technologies that combine NAIA with the CRISPR/Cas system, represented by SHERLOCK and DETECTR, have further improved the sensitivity of pathogen nucleic acid detection. However, these technologies require opening the reaction tube during the detection process, which is not only cumbersome but also poses a risk of aerosol contamination [11]. To address this issue, many researchers have been committed to developing more efficient one-step detection technologies. However, due to compatibility issues between NAIA and the CRISPR/Cas system, the CRISPR/Cas detection system competes with the NAIA initiation template, resulting in reduced sensitivity in one-step detection [20].

In this study, we developed a novel temperature-regulated physical isolation method for single-tube two-step detection, leveraging the physical state changes of tricosane at different temperatures. This method integrates isothermal amplification technology with CRISPR/Cas technology within a single tube, establishing a rapid, sensitive, and low-contamination-risk single-tube two-step MIRA-CRISPR/Cas12b assay. Tricosane exists as solid at room temperature and melts into liquid at 60 °C. However, tricosane becomes extremely viscous in its liquid state, necessitating very slow pipetting to aspirate precise volumes. Moreover, when handling a large number of reaction tubes over an extended period, the melted tricosane is prone to solidification, which can compromise experimental efficiency. To address this issue, we prefabricated tricosane into microspheres of precise volume. The prefabricated solid tricosane microspheres are more convenient to use and could be stored at room temperature long term. During the reaction setup, one microsphere is added to each tube, melted by heating to 60 °C, and then cooled to form a separation layer for the two-step reaction. This innovation significantly improves experimental efficiency.

The single-tube two-step MIRA-CRISPR/Cas12b assay consists of two stages. First, the target nucleic acid is amplified at 42 °C using the MIRA reaction. Subsequently, the CRISPR detection is performed at 60 °C using the thermostable AapCas12b (AapCas12b can bind to SgRNA at 60 °C, and its trans-cleavage activity is activated by the target, enabling it to cleave the fluorescent probe and release a fluorescent signal for detection). Tricosane is utilized to maintain a solid state within the isothermal amplification range of MIRA (37–42 °C), sealing the lower reaction system, and to transition to a liquid state within the high-temperature reaction range of CRISPR/Cas12b (58–65 °C), allowing the upper amplified product to enter the bottom of the reaction tube for the detection reaction. This approach physically separates the amplification and detection steps within a single tube. The entire detection process is completed in 50 min, compared to the 1.5 h required for qPCR, significantly reducing the detection time. Moreover, both the isothermal amplification and the CRISPR/Cas12b detection processes are conducted within a single tube without the need to open the tube or transfer liquids. Compared to the “capping-tube” single-tube assay proposed by Wang et al. [21], this method is more convenient in experimental operation, with an integrated detection workflow and a lower risk of aerosol contamination. In 2023, Du Dan’s team [22] developed a one-step RAA-CRISPR/Cas detection technology using an insert tube. By drilling a hole at the bottom of the insert tube, the Cas system was introduced into the tube with the RAA mixture placed at the bottom. After the RAA reaction was completed, centrifugation was performed to carry out the Cas cleavage reaction. However, this method requires pre-mixing of the RAA reagents. The pre-added magnesium can prematurely activate the DNA polymerase, affecting the reproducibility and accuracy of the experiment and limiting the reaction sensitivity [23,24]. The single-tube two-step assay with physical isolation developed in this study utilizes tricosane as a barrier to separate the isothermal amplification system from the CRISPR/Cas12b detection system. This approach prevents the reduction in reaction sensitivity caused by incompatibility between the two reaction systems. Moreover, by avoiding the need to pre-mix the isothermal amplification reagents, the risk of contamination is minimized while further enhancing reaction sensitivity. By optimizing the MIRA reaction time, reaction temperature, final primer concentration, tricosane volume, Cas12b volume, and the volume ratio of the MIRA amplification reaction to the CRISPR/Cas12b detection reaction, the optimal reaction conditions were determined. The LOD for MPXV using the single-tube two-step MIRA-CRISPR/Cas12b assay was found to be four copies per reaction. Compared to the traditional two-step MIRA-CRISPR/Cas12b assay, the LOD of 3.4 copies per reaction shows no significant difference. Compared to the two-step SHERLOCK [13] detection platform, which has an LOD of 10 copies per reaction, the sensitivity of the reaction was improved by a factor of 2.5. Compared to the qPCR method for MPXV established by Li et al. [25], which has an LOD of 10 copies per reaction, the sensitivity of the reaction was improved by a factor of 2.5. When applied to the detection of 100 clinical samples, the results showed that the single-tube two-step MIRA-CRISPR/Cas12b assay developed in this study exhibited good consistency with the qPCR assay (Kappa = 0.9547).

In this study, we designed the primers and sgRNA based on the conserved F3L fragment of the MPXV. However, the genomic sequences of different virus species within the genus Orthopoxvirus are similar, and cross-reactions may occur when different detection methods are applied [26]. Although the MIRA amplification primers, F1 and R1, we used have some mismatched sites with the genomic templates of non-Mpox virus species within the genus Orthopoxvirus, such as vaccinia virus, cowpox virus, variola virus, and Akhmeta virus, we are unable to determine whether primers F1 and R1 will cause a certain degree of nucleic acid amplification for these non-MPXV species within Orthopoxvirus. However, the CRISPR/Cas12b detection is also a specific detection process. Therefore, when designing the sgRNA, we particularly considered its specificity for the MPXV. For example, when the MPXV-SgRNA2 recognizes the target, the base “A” at the fifth position adjacent to the protospacer adjacent motif (PAM) site can only pair with the MPXV and cannot bind to other virus species within the genus Orthopoxvirus. Moreover, this position is crucial for the activation of the Cas enzyme. Therefore, theoretically, the MIRA-CRISPR/Cas12b detection method is specific for the detection of MPXV species. However, due to the lack of relevant clinical samples, this study has not verified other non-MPXV species within the genus Orthopoxvirus, and the species specificity of this detection method still requires further clinical validation in the future.

This study also has certain limitations. Firstly, the single-tube two-step MIRA-CRISPR/Cas12b assay developed here is based on nucleic acid extraction using a nucleic acid extractor, and the impact of different nucleic acid extraction methods on the detection results has not been verified. Secondly, the evaluation of the clinical sample detection performance of this technique was based on a relatively small sample size, which may affect the representativeness and comprehensiveness of the assessment results. Lastly, the current method is designed to detect a single target only. Future work could focus on developing the capability for simultaneous detection of multiple targets, enabling high-throughput on-site detection of multiple pathogens in complex samples, and thereby enhancing the application potential of this method in public health surveillance and outbreak response.

## 5. Conclusions

In conclusion, a highly sensitive and specific single-tube two-step MIRA-CRISPR/Cas12b assay has been developed for the rapid (within 45 min) detection of MPXV. This study also provides a basis for the development of similar detection methods for other infectious pathogens.

## Figures and Tables

**Table 1:** Sequences of primers, probes, and sgRNA for MPXV MIRA.

Primers/Probes	Sequence (5′-3′)
MPXV-MIRA-F1	CCTTATCGAATACTCTTCCGTCAATGTCTAC
MPXV-MIRA-R1	CTCTGTATGATCTTCAACGTAGTGCTATGG
MPXV-MIRA-F2	CTACAGCCAATTTAGCTGCATTATTTTTAGC
MPXV-MIRA-R2	CCGCGAAAAATCAATGAGAGAGGATCATAAG
MPXV-SgRNA1	GUCUAGAGGACAGAAUUUUUCAACGGGUGUGCCAAUGGCCACUUUCCAGGUGGCAAAGCCCGUUGAGCUUCUCAAAUCUGAGAAGUGGCACGTATTGAATCAGTGGGGCCT
MPXV-SgRNA2	GUCUAGAGGACAGAAUUUUUCAACGGGUGUGCCAAUGGCCACUUUCCAGGUGGCAAAGCCCGUUGAGCUUCUCAAAUCUGAGAAGUGGCACTTTAACCGGAATAACATCAT
MPXV-SgRNA1 transcription template	AGGCCCCACTGATTCAATACGTGCCACTTCTCAGATTTGAGAAGCTCAACGGGCTTTGCCACCTGGAAAGTGGCCATTGGCACACCCGTTGAAAAATTCTGTCCTCTAGACCCCTATAGTGAGTCGTATTA
MPXV-SgRNA2 transcription template	ATGATGTTATTCCGGTTAAAGTGCCACTTCTCAGATTTGAGAAGCTCAACGGGCTTTGCCACCTGGAAAGTGGCCATTGGCACACCCGTTGAAAAATTCTGTCCTCTAGACCCCTATAGTGAGTCGTATTA
FAM-Probe	FAM-TTTTT-BHQ

**Table 2:** Determination of the LOD for the single-tube two-step MIRA-CRISPR/Cas12b assay (fluorescence assays and visual assay) and the traditional two-step MIRA-CRISPR/Cas12b assay.

Copies/μL	Total Numbers	Fluorescence Assay	Visual Assay	Two-Step Assay
		Positive Numbers	Positive Numbers	Positive Numbers
10^4^	8	8	8	8
10^3^	8	8	8	8
10^2^	8	8	8	8
10^1^	8	8	8	8
10^0^	8	2	2	3
NC	8	0	0	0

**Table 3:** Comparison of the consistency between MIRA-CRISPR/Cas12b single-tube two-step assay and qPCR in detecting clinical samples.

Detection Method	qPCR		
	Positive	Negative	Total
MIRA-CRISPR/Cas12b			
positive	66	1	67
negative	1	32	33
total	67	33	100

## Data Availability

The data is contained within the article. The original contributions presented in this study are included in the article, and further inquiries can be directed to the corresponding authors.

## Abbreviations

The following abbreviations are used in this manuscript:

MPXV	Mpox Virus
MIRA	Multienzyme Isothermal Rapid Amplification
CRISPR/Cas	Clustered Regularly Interspaced Short Palindromic Repeats/CRISPR-Associated Protein
qPCR	Quantitative Real-Time PCR
LOD	Limit of Detection
NAT	Nucleic Acid Testing
NAIA	Nucleic Acid Isothermal Amplification
SHERLOCK	Specific High-Sensitivity Enzymatic Reporter Unlocking
DETECTR	DNA Endonuclease-Targeted CRISPR Trans Reporter
